# Desmocollin-2 inhibits cell proliferation and promotes apoptosis in hepatocellular carcinoma via the ERK/c-MYC signaling pathway

**DOI:** 10.18632/aging.204370

**Published:** 2022-11-08

**Authors:** Bo He, Li Guo, Youwen Hu, Hongyan Huang, Lijun Wan, Kedong Xu, Fenfen Wang, Zhili Wen

**Affiliations:** 1 Department of Gastroenterology, The Second Affiliated Hospital of Nanchang University, Nanchang, Jiangxi 330000, China

**Keywords:** desmocollin-2, proliferation, apoptosis, hepatocellular carcinoma, ERK/c-MYC

## Abstract

Hepatocellular carcinoma (HCC) is one of the most common cancers around the world with a poor prognosis. The main reason for poor prognosis is early stage HCC is inconspicuous so it is difficult to detect and effective treatment strategies are lacking for advanced HCC. In this context, novel molecular targets are urgently needed for the diagnosis and therapy of HCC. In this study, we investigated the expression level, biological function, and relative mechanism of Desmocollin-2(DSC2) in HCC. DSC2 expression levels were decreased significantly in HCC cell lines SMMC-7721(7721), Huh7, HCC-LM3(LM3), and MHCC-97H(97H), especially in LM3 cells, compared with human liver cell line L02(L02). DSC2 overexpression in LM3 cells could inhibit the proliferation (*in vitro* and *in vivo*), colony formation, migration, and invasion abilities of HCC cells, and promote cell apoptosis, while DSC2 inhibition in 7721 cells performed the opposite effect. Consistent with these results, regulating DSC2 expression in 7721 and LM3 cells could affect the expression levels of apoptosis-related proteins (Bax, Bcl-2, c-Caspase-3, Caspase-3, Caspase-8, and Survivin) and cell cycle-related proteins (Cyclin D1, Cyclin B1, CDK1, and CDK2). Furthermore, DSC2 expression was significantly negatively correlated with the levels of p-ERK and c-MYC in both LM3 and 7721 cell lines. These findings confirmed that DSC2 overexpression could inhibit the proliferation, migration, and invasion abilities while promoting apoptosis of HCC cells via the ERK/c-MYC signaling pathway. In a conclusion, DSC2 was a tumor suppressor with low expression in liver cancer.

## INTRODUCTION

Hepatocellular carcinoma (HCC) is one of the most common digestive system malignant tumors, which have contributed to high morbidity and mortality worldwide, especially in Asia and Africa [1–3]. As the early stage of HCC is often asymptomatic or mild symptoms, a majority of patients with HCC are diagnosed at an intermediate-advanced stage characterized by rapid invasion and metastasis, which is the major cause of death among patients with HCC [4]. At present, effective therapies for intermediate-advanced HCC patients are lacking, so it is imperative to explore new targets and possible molecular pathways for HCC treatment.

Loss of cell-cell adhesion is an initial event in the process of tumor spread and metastasis [5]. Desmosome is considered to be the most important junctional complex of intercellular adhesion in epithelial and some non-epithelial tissues [6]. Desmosome instability caused by desmosome deficiency may promote epithelial-mesenchymal transformation (EMT) and participate in tumor metastasis [7]. Desmocollin (DSC) is an important component of desmosome protein. There are three subtypes of the DSC family, namely Desmocollin-1(DSC1), Desmocollin-2(DSC2), Desmocollin-3(DSC3), and four subtypes of Desmoglein (DSG1-4), which constitute the desmosome cadherin family [8]. DSC2 is an important member of the DSC family and is widely distributed in all desmosome-containing tissues [9]. However, DSC2 is dysregulated in many diseases. Several studies have reported that DSC2 is associated with many kinds of pemphigus, and its antibody can be detected in the serum of patients [10–12]. DSC2 has also been shown to be dysregulated in human arrhythmogenic right ventricular dysplasia/cardiomyopathy [13–15]. In addition, DSC2 is involved in the mechanism of injury and repair of the intestinal mucosa. Down-regulation of DSC2 can weaken cell-matrix traction, decrease the levels of integrin β 1 and β 4, change the activity of small GTPase Rap1, and ultimately affect the migration and repair of intestinal epithelial cells [16]. Recently, studies have found that the expression of DSC2 is abnormal in many human cancers, and it is related to the prognosis of tumors. The abnormal expression forms in various cancers may be different. For example, the expression of DSC2 was up-regulated in prostate cancer [17], while down-regulated in esophageal carcinoma [18, 19], gastric cancer [20], pancreatic ductal adenocarcinoma [21], colorectal cancer [22], urothelial carcinoma [23] and lung cancer [24]. However, these studies all suggested that the abnormal expression of DSC2 can play essential roles in tumorigenesis and development.

At present, evidence suggests abnormal DSC2 expression affecting tumorigenesis and development is mainly related to β-catenin/γ-catenin signaling pathways. The abnormal expression of DSC2 reversibly regulates the localization of adhesion junction molecules on the cell membrane. Down-regulation of DSC2 mediated by miR-25 promotes the proliferation and invasiveness of cancer cells by redistributing β-catenin and activating the β-catenin signaling pathway [19]. Loss of DSC2 can activate Akt signaling pathway and promote tumor proliferation [25]. Additionally, the level of EGFR increases after the loss of DSC2, which helps to release γ-catenin translocation from desmosome to nucleus, thus stimulating the transcription of c-MYC or Bcl-2 and promoting cell proliferation [25]. It is noteworthy that the ERK pathway is a classical signaling pathway that regulates tumor proliferation and apoptosis, etc. At present, whether DSC2 can affect the ERK pathway to regulate tumor proliferation and apoptosis has not been reported, and there remains unknown about the roles of DSC2 in the initiation and progression of HCC. So, we systematically investigated whether aberrant DSC2 expression can influence HCC development by modulating proliferation, apoptosis, migration, and invasion via the ERK pathway. In conclusion, this study is expected to provide a new theoretical foundation and strategy for the treatment of HCC in the future.

## RESULTS

### DSC2 expression levels in HCC cell lines

**Figure 1:**
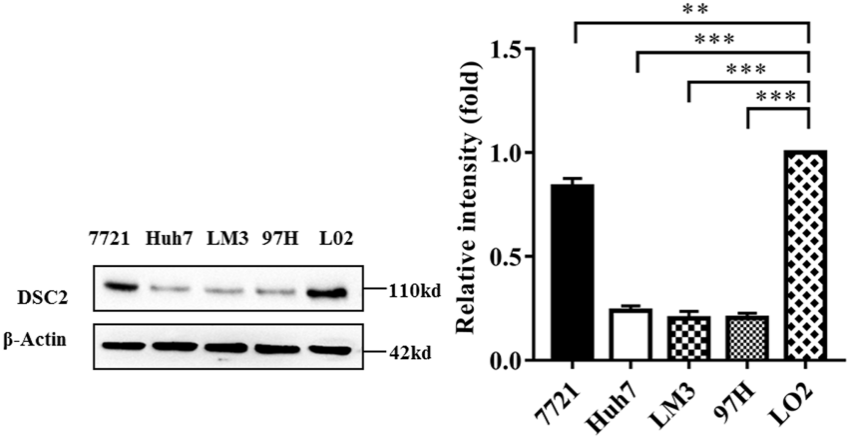
**DSC2 expression in HCC cell lines.** DSC2 expression levels in L02 and HCC cell strains were detected by Western blot analysis (repeated three times). ^*^P < 0.05, ^**^P < 0.01, ^***^P < 0.001.

To understand the expression of DSC2 in HCC cell lines, we detected the expression of DSC2 in normal hepatocyte cells (L02) and HCC cell lines (SMMC-7721, Huh7, HCC-LM3, and MHCC-97H). The results showed that the expression of DSC2 protein in HCC cell lines SMMC-7721 (*P* < 0.01), Huh7 (*P* < 0.001), HCC-LM3 (*P* < 0.001), and MHCC-97H (*P* < 0.001) were all significantly down-regulated compared with L02 cells (Figure 1). In addition, in the HCC cell lines above, DSC2 was highly expressed in 7721 cells, while the expression level was the lowest in LM3 cells (highly metastatic cell line). So we chose 7721 and LM3 for subsequent experiments.

### Selecting overExp-DSC2 and shRNA-DSC2-1 plasmids for transfections

Since the expression of DSC2 in LM3 cells was lower than that in Huh7, 97H, and 7721 cells, we overexpressed DSC2 in LM3 cells and interfered with the expression of DSC2 in 7721 cells. We found that after transfection with an overexpression vector (overExp-DSC2), the expression of DSC2 in LM3 cells was significantly up-regulated (*P* < 0.05). Then, we confirmed that the expression of DSC2 in 7721 cells had successfully interfered with the specific shRNA (shRNA-DSC2-1 and shRNA-DSC2-2) (*P* < 0.001), while the expression level of DSC2 in 7721 cells transfected with the shRNA-DSC2-3 vector was not significantly altered (*P* > 0.05) (Supplementary Figure 1). Because of the lowest expression of DSC2 in cells after shRNA-DSC2-1 interference, the shRNA-DSC2-1 plasmid was selected for subsequent experiments.

### DSC2 inhibited the ability of cell proliferation

**Figure 2:**
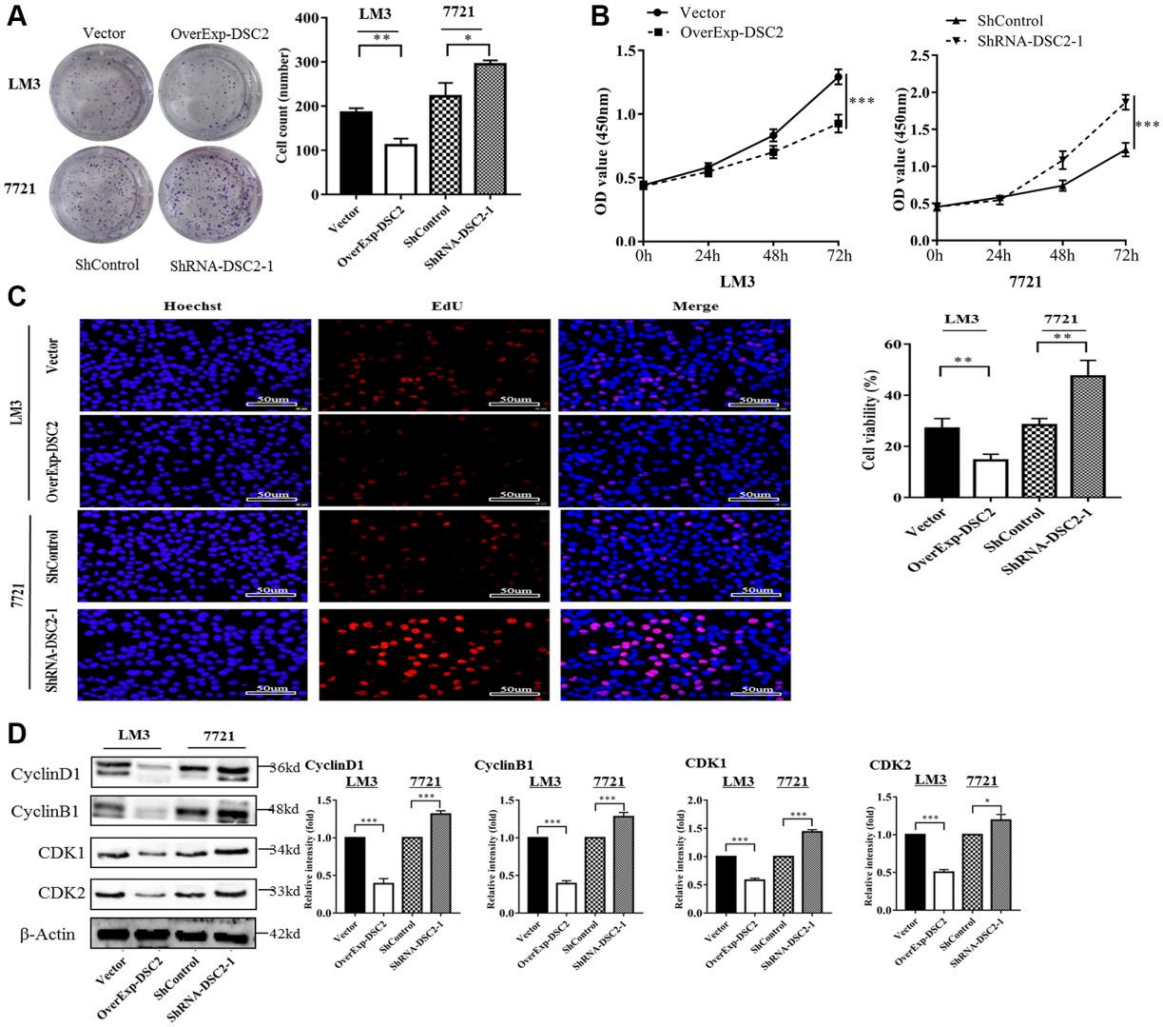
**DSC2 inhibits the cell proliferation ability *in vitro* and affects cell cycle-related proteins.** (**A**) The clone formation ability of overExp-DSC2-transfected LM3 and shRNA-DSC2-1-transfected 7721 cells was shown by clone formation assay (repeated three times). ^*^P < 0.05, ^**^P < 0.01, ^***^P < 0.001. (**B**, **C**) The cell viability of overExp-DSC2-transfected LM3 and shRNA-DSC2-1-transfected 7721 cells were detected by CCK-8 and EdU assay (repeated three times for both). ^*^P < 0.05, ^**^P < 0.01, ^***^P < 0.001. (**D**) Western blot analysis of the protein levels of Cyclin D1, Cyclin B1, CDK1, and CDK2 from LM3 and 7721 cells transfected with overExp-DSC2 and shRNA-DSC2-1 (repeated three times). ^*^P < 0.05, ^**^P < 0.01, ^***^P < 0.001.

To explore the effect of DSC2 on cell proliferation, we detected the proliferation ability of transfected cells by using colony formation, CCK-8, and EdU assays. Results indicated that the formation of tumor cell colonies was significantly inhibited in overExp-DSC2-transfected LM3 cells (*P* < 0.01), while promoted in shRNA-DSC2-transfected 7721 cells (*P* < 0.05) (Figure 2A). Similarly, CCK8 assay showed that there were significantly lower rates of cell proliferation in LM3 cells with overExp-DSC2, but higher proliferation rates in 7721 cells with shRNA-DSC2 (Figure 2B, *P* < 0.001). We further verified the effect of DSC2 on proliferation by using the EdU experiment. It was also found that the proliferation capacity of the overExp-DSC2-transfected LM3 cells was noticeably decreased, whereas it was increased for 7721 cells transfected with shRNA-DSC2 (Figure 2C, *P* < 0.01).

To understand the effect of DSC2 on the cell cycle, we detected the expression levels of cell cycle-related proteins in transfected cells by Western blotting and the results demonstrated that the expression levels of Cyclin D1, Cyclin B, CDK1, and CDK2 were significantly down-regulated in overExp-DSC2-transfected LM3 cells (*P* < 0.001), while up-regulated in shRNA-DSC2-transfected 7721 cells (*P* < 0.05) (Figure 2D). These results indicated that DSC2 overexpression suppressed HCC cell proliferation, while DSC2 knockdown promoted HCC cell proliferation.

### DSC2 promoted cell apoptosis and regulated apoptosis-related proteins expression

Transfected cells were stained with Acridine Orange/Ethidium Bromide (AO/EB) to detect cell apoptosis, and the results indicated that DSC2 overexpression significantly increased apoptosis in overExp-DSC2-transfected LM3 cells (*P* < 0.001), while the knockdown of DSC2 decreased apoptosis in shRNA-DSC2-transfected 7721 cells (*P* < 0.01) (Supplementary Figure 2A).

To further clarify the role of DSC2 on cellular apoptosis, we assessed the expression of key apoptosis-related proteins in the transfected cells. The results showed that the expression levels of Bax, c-Caspase-3, and Caspase-8 were noticeably increased in overExp-DSC2-transfected LM3 cells (*P* < 0.01), whereas the expression levels of Bcl-2 and Survivin were decreased (*P* < 0.01). Furthermore, the expression levels of Bcl-2 (*P* < 0.001) and Survivin (*P* < 0.05) were elevated in shRNA-DSC2-transfected 7721 cells, while the expression levels of Bax and c-Caspase-3 were reduced significantly (*P* < 0.001) (Supplementary Figure 2B). In conclusion, DSC2 overexpression could promote apoptosis of HCC cells.

### DSC2 inhibited HCC cells migration and invasion

To investigate whether the DSC2 affected the migration and invasion of HCC cells, cell wound healing and Transwell assays were performed. Wound healing assays demonstrated that DSC2 overexpression in LM3 cells inhibited the cell motility (*P* < 0.05 for 24 h and *P* < 0.01 for 48 h), while DSC2 knockdown exhibited the opposite effect in 7721 cells (*P* < 0.05 for 24 h and *P* < 0.01 for 48 h) (Supplementary Figure 3A). Moreover, Transwell assay showed that the migration and invasion abilities were decreased dramatically in overExp-DSC2-transfected LM3 cells (*P* < 0.01), while increased in shRNA-DSC2-transfected 7721 cells (*P* < 0.05) (Supplementary Figure 3B). These findings suggested that DSC2 overexpression could inhibit the cell migration and invasion abilities of HCC cells and vice versa.

### DSC2 expression levels affected the ERK/c-MYC signaling pathway

**Figure 3:**
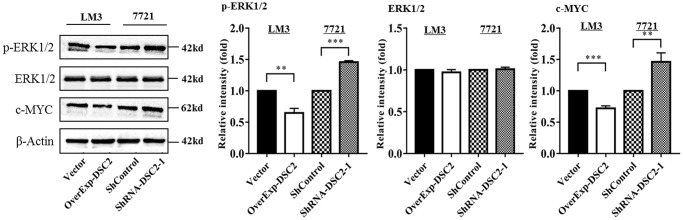
**DSC2 inhibits the ERK1/2 signaling pathway.** The expression levels of ERK1/2, p-ERK1/2, and c-MYC in overExp-DSC2-transfected LM3 and shRNA-DSC2-1-transfected 7721 cells were determined by Western blot analysis (repeated three times). ^*^P < 0.05, ^**^P < 0.01, ^***^P < 0.001.

To explore the molecular mechanism of DSC2 inhibiting proliferation and promoting apoptosis in HCC cells, we detected the ERK1/2 signaling pathway. The results showed that the overexpression of DSC2 inhibited the expression of p-ERK1/2 (p-ERK1-T202/Y204+p-ERK2-T185/Y187) and c-MYC in LM3 cells, while DSC2 knockdown increased the expression levels of p-ERK1/2 and c-MYC in 7721 cells (Figure 3). These data indicate that DSC2 may affect the proliferation, apoptosis, migration, and invasion of HCC cells by regulating the ERK/c-MYC signaling pathway.

### DSC2 inhibited the growth of tumors *in vivo*

**Figure 4:**
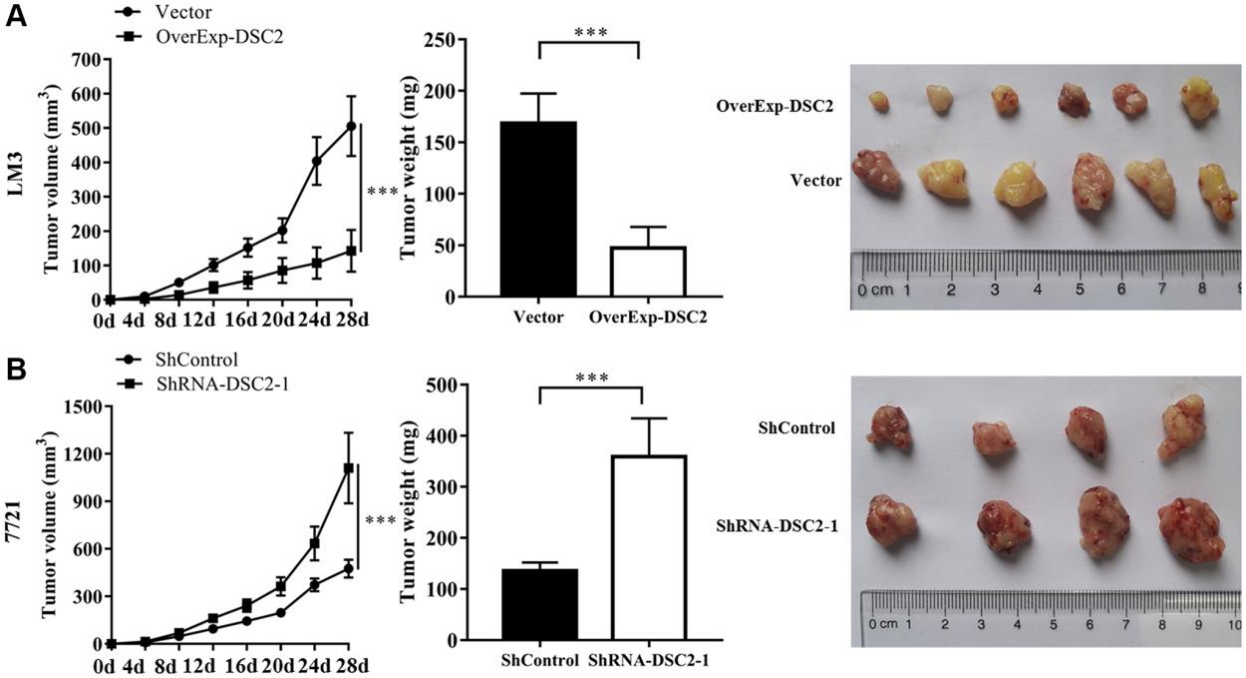
**DSC2 inhibits HCC tumor growth *in vivo*.** (**A**) LM3 cells transfected with vector and overExp-DSC2 were injected into the right back of 5-week-old male nude mice with 6 rats in each group. After 28 days, the tumor was collected, and the volume and weight of the tumor were measured. (**B**) 7721 cells transfected with shControl and shRNA-DSC2-1 were injected into the right back of 5-week-old male nude mice with 6 rats in each group. After 28 days, the tumor was collected, and the volume and weight of the tumor were measured.

To identify in-depth the effect of DSC2 on tumor growth *in vivo*, the transfected cells (vector, overExp-DSC2, shControl, and shRNA-DSC2 respectively) were subcutaneously injected into the right back of 5-week-old nude mice. Tumor volume was measured and recorded every 4 days. Mice were sacrificed 28 days later for sampling. Subsequently, tumor volume and weight were determined. As shown in Figure 4A, the volume and weight of the tumors from the mice injected with DSC2-overexpressing LM3 cells were significantly lower than those from the mice injected with the vector-transfected cells (*P* < 0.001 for both), indicating that DSC2 overexpression inhibited the tumor growth dramatically. Additionally, as shown in Figure 4B, we found that the volume and weight of tumors in mice injected with the DSC2-knockdown cells were markedly higher than those in the mice injected with shControl-transfected cells (*P* < 0.001 for both), indicating the down-regulation of DSC2 promoted the tumor growth.

## DISCUSSION

Cell adhesion is a key factor in maintaining the normal structure and function of epithelial tissue [26]. Aberrant expression and function of adhesion proteins were closely associated with tumorigenesis and development [19, 27]. DSC2, a subtype of the desmocollin family, participates in epithelial cell-cell adhesion and the stabilization of normal tissue structure as a key role [28, 29]. Previous studies have verified that several desmosomal proteins are dysregulated in diverse human cancers, but the role of DSC2 in HCC has not been investigated so far [25, 30, 31]. Our study showed that the expression levels of DSC2 were significantly down-regulated in HCC cells compared with that in the human liver cell line (L02), especially in LM3 cells. This is consistent with previous reports in other cancers. This suggested that DSC2 may play a role in the development of HCC.

DSC2 participates in cell signal transduction and mediates a variety of biological processes, including cell adhesion, apoptosis, proliferation, migration, and invasion [17, 25, 31, 32]. Low levels of DSC2 expression could lead to bradycardia, ventricular dilatation, abnormal cardiac contractility, and cardiomyocyte edema [33]. Double down-regulation of DSC2 and DSG2 saved the down-regulation of DSG2 alone on the growth inhibition of colon cancer cell lines, suggesting that down-regulation of DSC2 could promote the proliferation of colon cancer cells [34]. Consistent with our speculation, our current study clearly showed that overexpression of DSC2 could inhibit the proliferation and promote apoptosis of HCC cells, while inhibition of DSC2 exhibited the opposite effect. This result was also confirmed *in vivo*. Consistent with the changes in proliferation and apoptosis, the expression levels of cell cycle-related proteins and apoptosis-related proteins were significantly affected by DSC2 expression [17].

Invasion and metastasis are the two most important signs of malignant tumors, as well as the most important predictors of the lethality of human cancers [35]. More and more evidence showed that DSC2 plays a crucial role in the metastatic spread of tumors [17, 19, 36]. In tumors, down-regulation of DSC2 led to cell adhesion defect, reduces desmosome expression level and adhesion junction molecule distribution, and inhibits keratin intermediate filament retraction and filamentous actin cytoskeleton rearrangement, resulting in the increased migratory and invasive abilities of DSC2-deficient tumor cells [32]. In agreement with previous studies, our data showed that overexpression of DSC2 could inhibit the migration and invasion of HCC cells, and knockdown of DSC2 presented the opposite effect.

Activation of ERK phosphorylates other transcription factors and binds to promoters such as growth factors and cytokine genes, thereby promoting cell growth and inhibiting apoptosis [37, 38]. Proto-oncogene c-MYC is an important member of the MYC gene family, which participates in cell metabolism, proliferation, and differentiation [39]. Proto-oncogene c-MYC plays an important role in the carcinogenic transformation of normal cells and tumor proliferation. Down-regulation of c-MYC could significantly inhibit the growth of HCC cells [40, 41]. However, the association between DSC2 and the ERK1/2 pathway has not been reported so far. Therefore, we are the first to explore the relationship between DSC2 and the ERK/c-MYC pathway. Consistent with the prediction, our study showed that up-regulation of DSC2 inhibited the expression levels of p-ERK1/2 and c-MYC in HCC cells. Correspondingly, the down-regulation of DSC2 promoted the expression levels. The results suggested that DSC2 may regulate the proliferation, apoptosis, migration, and invasion of HCC cells via the ERK/c-MYC signaling pathway.

This study was beset by some limitations. Firstly, due to the limited funds and time, our current study mainly focused on cell and animal experiments, rather than clinical experiments for verification, which we expect to perform in the follow-up study. Secondly, in this study, the mechanism of action of DSC2 affecting HCC cells on proliferation and apoptosis was not in-depth enough.

In conclusion, our study indicated for the first time that the expression levels of DSC2 were lower significantly in HCC cells than in human liver cells, and overexpression of DSC2 strongly inhibited the proliferation, migration, and invasion, while promoting apoptosis in HCC cells. Correspondingly, the knockdown of DSC2 promoted proliferation, migration, and invasion, while suppressing apoptosis. Additionally, it was confirmed that DSC2 overexpression significantly inhibited HCC tumor growth *in vivo.* The function of DSC2 may be triggered by activating the ERK/c-MYC pathway. Our study, for the first time, provided an understanding of the potential effect and mechanism of DSC2 on HCC cells. These findings suggest that DSC2 may be a novel biomarker and a potential therapeutic target of HCC in the future.

## MATERIALS AND METHODS

### Cell culture and transfection

HCC cell lines SMMC-7721, Huh7, HCC-LM3 (highly metastatic cell line), MHCC-97H (highly metastatic cell line), and human immortalized hepatocyte line L02 obtained from Chinese Academy of Sciences Cell Bank (Chinese Academy of Sciences, Shanghai, China) were identified by STR and detected by mycoplasma. The cells were cultured in high glucose DMEM (Invitrogen, CA, USA), which was supplemented with 100 U/ml penicillin, 100 U/ml streptomycin, and 10% fetal bovine serum (FBS). The cells were cultured in 37°C, 5% CO_2_ incubator, and had been subcultured after digestion with 0.05% EDTA-trypsin. The cells in the logarithmic growth phase were inoculated in a 6-well plate with 2 × 10^4^ cells/well. 16–18 hours later, the cells were transfected with vector, overExp-DSC2, shControl, shRNA-DSC2-1, shRNA-DSC2-2, and shRNA-DSC2-3 (Shanghai Jikai Gene Co., Ltd., Shanghai, China). Puromycin (2 ug/ml) was used to screen the cell lines. The expression of DSC2 was detected by RT-qPCR and Western blot to determine the transfection effect.

### Colony formation assay

The transfected cells were seeded into a 6-well plate with 500 cells/well for routine culture. The cells were cultured for 2–3 weeks and terminated when visible cell clones emerged in the 6-well plate. Washed twice with PBS, the clones were fixed with 4% paraformaldehyde for 15 min and then stained with Wright-Giemsa compound staining solution for 30 min. After washing and drying, the clones were observed and photographed.

### Cell counting kit-8 (CCK-8)

The transfected cells were collected and inoculated into 96-well plates (8000 cells/well) for routine culture. After the cells adhered to the wall, 10 ul CCK-8 (Yuheng Biotechnology Co., Ltd; Jiangsu, China) was added at 0 h, 24 h, 48 h, and 72 h, respectively, and incubated at 37°C for 2 hours in dark. The optical density (OD) at 450 nm was measured by Thermo Multiskan FC (Thermo Fisher, MA, USA).

### Edu assay

To determine the rate of cell proliferation, we visualized the incorporation of the thymidine analog 5-Ethynyl-2′-deoxyuridine (EdU) into newly synthesized DNA using the Click-it EdU kit (Yuheng Biotechnology Co., Ltd; Jiangsu, China). Cells were seeded on 96-well plates (1 × 10^4^ cells/well) and processed according to the instructions of the EDU reagent the next day. After Hoechst re-staining of nuclear for 5 min, it was observed and photographed under the fluorescence microscope. Counting was performed automatically using ImageJ (https://www.nih.gov).

### Subcutaneous tumorigenesis

This experiment has been approved by the Ethics Committee of the second affiliated Hospital of Nanchang University. Transfected cells (4 × 10^6^ cells/0.1 mL) were subcutaneously inoculated into the right back of 5-week-old nude mice, six mice in each group. Tumor volume was measured every 4 days. After tumor-bearing mice were sacrificed 28 days later, tumor tissue was harvested and weighed, and the tumor volume was measured.

### Wound healing assay

The transfected cells were seeded in a 6-well plate for conventional culture. After the cells were fused completely, a 10 μl pipette tip was used to scratch in a straight line vertically at the bottom of the wells. The monolayer cell fragments and residual medium were washed off by PBS, and the scratches were observed and photographed under the microscope at 0 h, 24 h, and 48 h, respectively. ImageJ (https://www.nih.gov) was used to quantify the degree of wound closure at the above-specified time points.

### Transwell assay

The bottom of the chamber was evenly smeared with a matrix to form a film. The transfected cells were collected and re-suspended in a serum-free DMEM medium to prepare a single-cell suspension. The upper chambers (no matrix glue/matrix glue) were added with 200 ul cell suspension containing 1 × 10^5^/2 × 10^5^ cells respectively, and 750 μl DMEM medium containing 10% fetal bovine serum was added into the lower chamber. The upper chamber was taken out and the culture medium was discarded after continuous culture for 24 hours. The chamber was fixed in 4% paraformaldehyde for 10 min, stained with 10 g/l crystal violet for 30 min, and gently wiped the cells on the upper chamber’s inner surface with a cotton swab. The cells were observed and photographed by the microscope.

### Acridine orange/ethidium bromide (AO/EB) assay

The transfected cells were collected and inoculated into a 96-well plate (1 × 10^4^ cells/well) for routine culture. The next day, the cells were incubated with 200 ul staining buffer and 2 ul AO/EB staining solution at room temperature for 30 min. The cells were observed and photographed by the fluorescence microscope after washing.

### Western blot analysis

As the transfected cells reached 90% fusion, the mixture of RIPA and protease inhibitor (400 ul) was added to the cells for 30 minutes (always performed on ice). The liquid was placed into an Eppendorf tube and centrifuged at 12,000 g for 15 min at 4°C. Protein concentration was estimated using the BCA protein assay kit (Beyotime Biotech, China). Equal amounts of protein (30 μg/lane) were resolved by 8% SDS-PAGE and transferred onto PVDF membranes. The membrane was then washed with Tris-buffered saline containing 0.05% Tween-20 (TBST) and blocked in TBST containing 5% non-fat dry milk or 5% BSA, and further incubated with the respective specific antibodies to DSC2, Bax, Bcl-2, Caspase-3, c-Caspase-3, Caspase-8, Survivin, Cyclin D1, Cyclin B1, CDK1, CDK2, ERK1/2, p-ERK1/2 (p-ERK1-T202/Y204+p-ERK2-T185/Y187) and c-MYC and β-actin. The membranes were then incubated with appropriate horseradish peroxidase (HRP)-conjugated secondary antibodies and developed using ECL western blotting detection reagents (Yuheng Biotechnology Co., Ltd; Jiangsu, China). All experiments were repeated three times.

### Statistical analyses

The statistical analysis was performed by using SPSS 25.0 (SPSS, Inc., Chicago, IL, USA). All data were expressed as the mean ± standard deviation. Comparisons between data sets were performed using the χ^2^ test and a two-tailed independent sample *t*-test when appropriate. *P* value < 0.05 was considered significant difference.

### Data availability statement

The original contributions presented in the study are included in the article/Supplementary Material, further inquiries can be directed to the corresponding authors.

## Supplementary Materials

Supplementary Figures
