# Supplementary material for: Desmocollin-2 inhibits cell proliferation and promotes apoptosis in hepatocellular carcinoma via the ERK/c-MYC signaling pathway
Source: Aging (Albany NY). 2022 Nov 8;14(21):8805–17. doi: 10.18632/aging.204370 (PMC9699757; doi:10.18632/aging.204370)
Supplement: Supplementary Figures [file aging-14-204370-s001.pdf]

SUPPLEMENTARY FIGURES

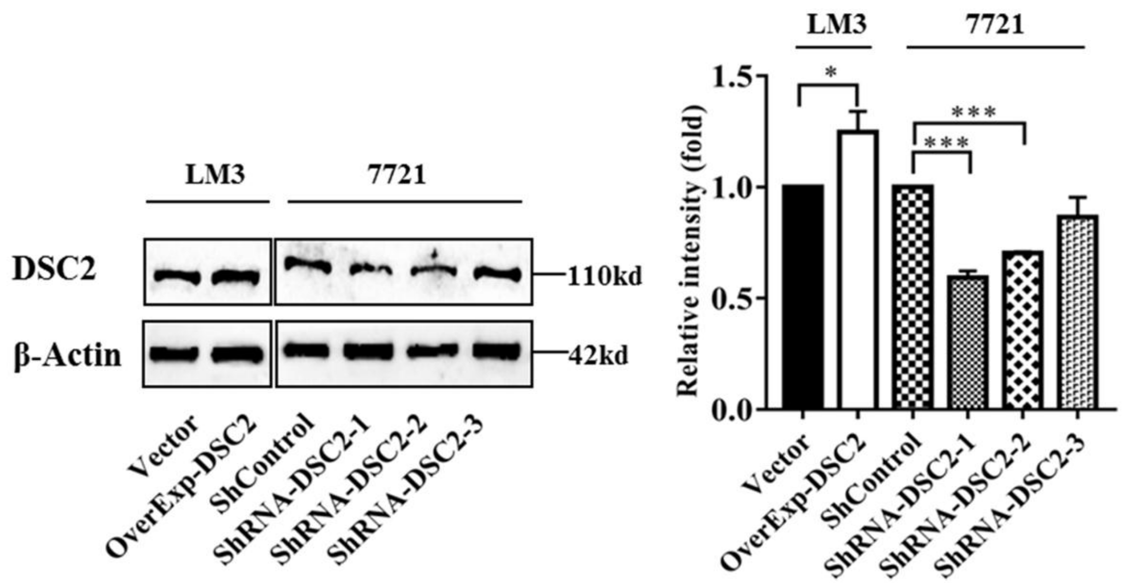

Supplementary Figure 1. OverExp-DSC2 and shRNA-DSC2-1 plasmids were chosen. DSC2 protein levels in LM3 and 7721 cells transfected with vector, overExp-DSC2, shControl, shRNA-DSC2-1, shRNA-DSC2-2, and shRNA-DSC2-3 were detected by Western blot analysis (repeated for three times). $^{*}P < 0.05$, $^{**}P < 0.01$, $^{***}P < 0.001$.

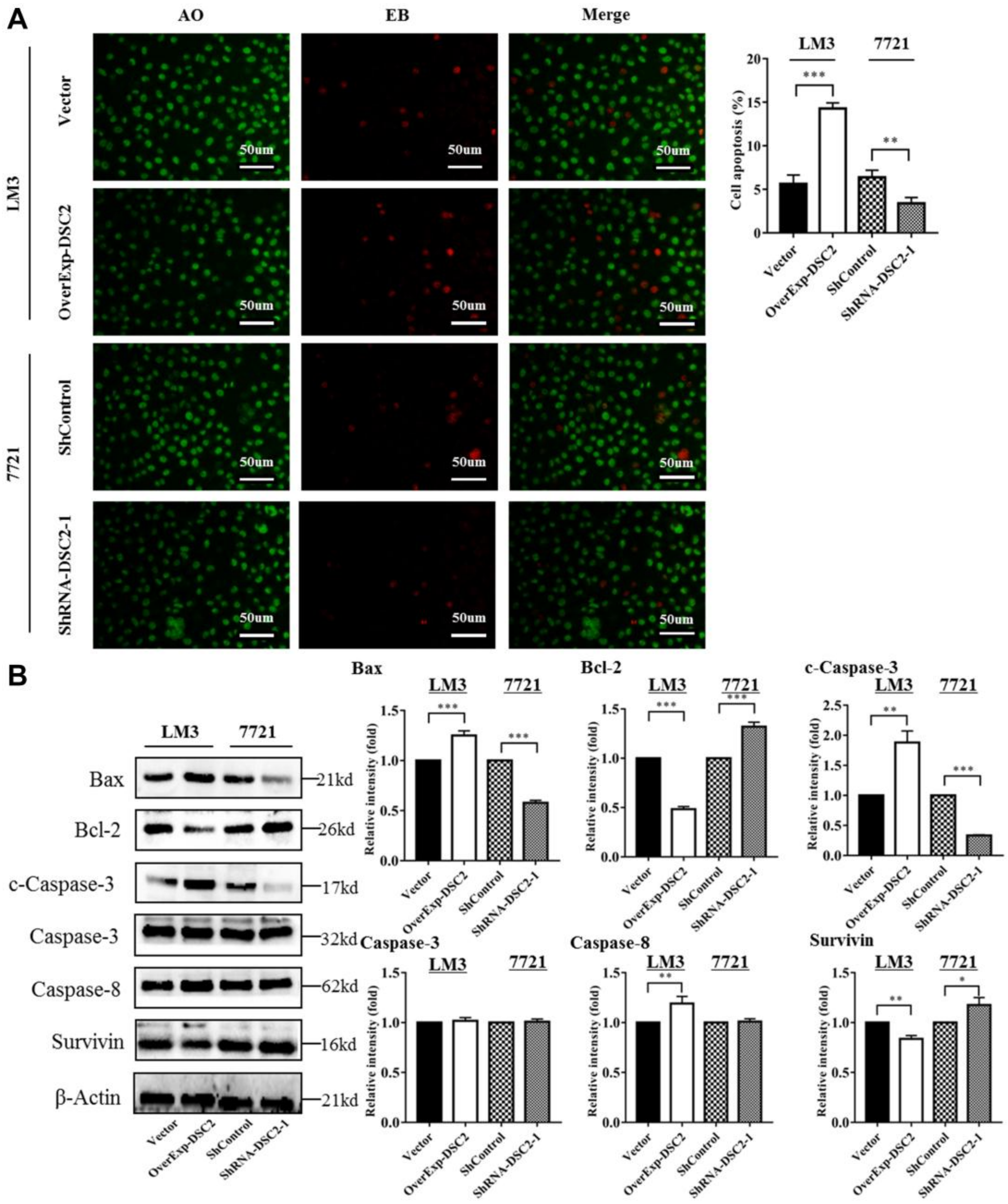

Supplementary Figure 2. DSC2 promotes cell apoptosis and affects apoptosis-related protein expression. (**A**) The apoptosis rate of overExp-DSC2-transfected LM3 and shRNA-DSC2-1-transfected 7721 cells were analyzed by Acridine Orange/Ethidium Bromide(AO/EB) assay (repeated three times). $^{*}P < 0.05$, $^{**}P < 0.01$, $^{***}P < 0.001$. (**B**) The expression levels of Bax, Bcl-2, c-Casp-3, Casp-3, Casp-8, and Survivin in overExp-DSC2-transfected LM3 and shRNA-DSC2-1-transfected 7721 cells were detected by Western blot analysis (repeated for three times). $^{*}P < 0.05$, $^{**}P < 0.01$, $^{***}P < 0.001$.

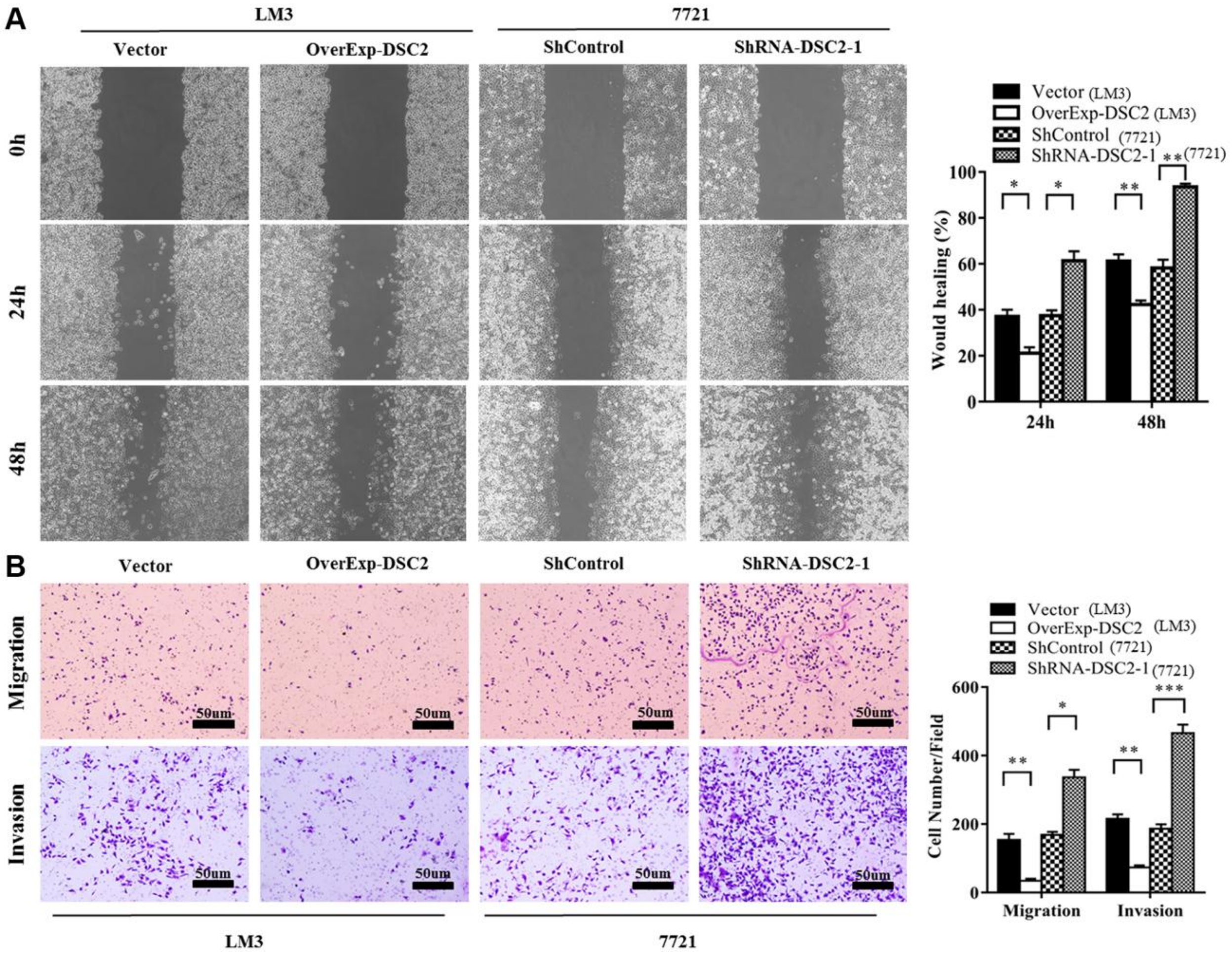

Supplementary Figure 3. DSC2 inhibits migration and invasion in HCC cells. The migration and invasion abilities of overExp-DSC2-transfected LM3 and shRNA-DSC2-1-transfected 7721 cells were respectively detected by wound healing assay (**A**) and transwell assay (**B**) (repeated three times for both). $^{*}P < 0.05$, $^{**}P < 0.01$, $^{***}P < 0.001$.
